# Mathematical modeling of intraperitoneal drug delivery: simulation of drug distribution in a single tumor nodule

**DOI:** 10.1080/10717544.2016.1269848

**Published:** 2017-02-09

**Authors:** Margo Steuperaert, Giuseppe Falvo D’Urso Labate, Charlotte Debbaut, Olivier De Wever, Christian Vanhove, Wim Ceelen, Patrick Segers

**Affiliations:** 1 Biofluid, Tissue and Solid Mechanics for Medical Applications (bioMMeda), Department of Electronics and Information Systems, iMinds Medical IT Department, Ghent University, Ghent, Belgium,; 2 Department of Environmental and Chemical Engineering, University of Calabria, Rende, CS, Italy,; 3 Department of Radiation Oncology and Experimental Cancer Research,; 4 Infinity (iMinds-IBiTech-MEDISIP), Department of Electronics and Information Systems, and; 5 Department of Surgery, Cancer Research Institute Ghent (CRIG), Ghent University, Ghent, Belgium

**Keywords:** Drug transport, intraperitoneal chemotherapy, carcinomatosis, computational fluid dynamics

## Abstract

The intraperitoneal (IP) administration of chemotherapy is an alternative treatment for peritoneal carcinomatosis, allowing for higher intratumor concentrations of the cytotoxic agent compared to intravenous administration. Nevertheless, drug penetration depths are still limited to a few millimeters. It is thus necessary to better understand the limiting factors behind this poor penetration in order to improve IP chemotherapy delivery. By developing a three-dimensional computational fluid dynamics (CFD) model for drug penetration in a tumor nodule, we investigated the impact of a number of key parameters on the drug transport and penetration depth during IP chemotherapy. Overall, smaller tumors showed better penetration than larger ones, which could be attributed to the lower IFP in smaller tumors. Furthermore, the model demonstrated large improvements in penetration depth by subjecting the tumor nodules to vascular normalization therapy, and illustrated the importance of the drug that is used for therapy. Explicitly modeling the necrotic core had a limited effect on the simulated penetration. Similarly, the penetration depth remained virtually constant when the Darcy permeability of the tissue changed. Our findings illustrate that the developed parametrical CFD model is a powerful tool providing more insight in the drug transport and penetration during IP chemotherapy.

## Introduction

Patients with peritoneal carcinomatosis suffer from a widespread metastatic growth of tumor nodules in the peritoneal cavity. This disease often originates from ovarian or colon carcinoma, and prognosis and expected quality of life is usually poor with a 5-year survival rate of less than 40% for advanced stage ovarian cancer and 12.5% for colorectal cancer (Burges & Schmalfeldt, 2011; Favoriti et al., 2016). Conventional intravenous (IV) chemotherapy does not offer a substantial improvement in the prognosis of the patients, while being very demanding on the patients due to its side-effects. The intraperitoneal (IP) administration of chemotherapy is an alternative treatment that allows for higher intratumor concentrations of the cytotoxic agent compared to IV administration, while maintaining the same plasma concentrations (Miyagi et al., 2005). When the chemotherapeutic agent is administered IP, the tumor nodule surfaces are in direct contact with the drug solution. In contrast, in IV delivery the drug is first transported convectively through the bloodstream, after which it extravasates through the microcirculation and finally penetrates the tumor tissue via diffusion and convective transport. Although IP chemotherapy is a promising technique, its actual clinical application is still limited due to the poor drug penetration (typically no more than a few millimeters) in the tumor tissue (Los et al., 1990; Royer et al., 2012; Ansaloni et al., 2015).

Drug penetration into solid tumors is a complex process that involves multiple parameters not only related to the used cytotoxic agent (e.g. diffusivity), but also to the tumor tissue properties (e.g. permeability) and even the therapeutic set-up (e.g. concentration). Like many solid tumors, the peritoneal tumor nodules often exhibit a high interstitial fluid pressure (IFP). This high IFP is caused by a number of contributing factors, including the leaky and irregularly shaped microvasculature, the lack of a functional lymphatic system, a denser extracellular matrix, an increased number of cancer associated fibroblasts (CAFs) and a larger cell density (Heldin et al., 2004). The net effect of all these factors is a radially outward pressure gradient and convective flux in the interstitium, as fluid flows toward the outer layers of the tumor (Jain et al., 1991). The latter effectively obstructs the diffusive penetration of the drug from the outer edge to the center of the tumor during IP chemotherapy. Due to the imbalance between supply and demand of oxygen and nutrients in the rapidly growing tumor, the majority of the tumor nodules have a necrotic core in which no viable cells or functional vascular system are located and, hence, no blood flow or cellular drug uptake is present. Additionally, not all of the drug that penetrates the tumor tissue will enter the cancer cells, as a part of the drug will be resorbed by the tumor microvasculature and convectively transported throughout the systemic circulation, or can be lost due to binding to the extracellular matrix. These processes further limit the amount of free drug that is available for deeper tissue penetration.

Drug tissue penetration is influenced by multiple parameters; the use of computational fluid dynamics (CFD) modeling has the benefit of being able to change single parameters and study their relative influence on the therapeutic outcome of the treatment without influencing other parameters. Changing some of the essential parameters over a well-defined region of interest allows for a better understanding of their impact on treatment outcome and thus allows for the optimization of drug transport during IP chemotherapy.

Previously, a number of modeling studies focused on the classical IV delivery of drugs and parameters of importance were vascular supply, drug release and activation, drug diffusive transport, drug advective transport and drug decay, deactivation and cellular uptake (Kim et al., 2013). Recently, CFD has been used to study a combination of these models whereby the implemented equations are usually based on the groundwork done by Baxter & Jain (1989). Substantial CFD modeling efforts have been done mainly in the area of brain tumors with a special focus on the comparison of different drug release systems (Kalyanasundaram et al., 1997; Wang & Li, 1998; Wang et al., 1999; Tan et al., 2003; Linninger et al., 2008; Arifin et al., 2009) and the incorporation of transient flow due to edema (Teo et al., 2005). A two-dimensional (2D) model, in which the delivery of doxorubicin to a hepatoma segmented of a patient CT-scan was simulated, included the extracellular binding and DNA binding of the drug (Goh et al., 2001). More parametric CFD models on solid tumors in general have been developed to study the influence of tumor size (Soltani & Chen, 2011), shape (Soltani & Chen, 2012) and the effect of different therapeutic options such as vascular normalization therapy (Ozturk et al., 2015) or thermosensitive drug delivery (Zhan & Xu, 2013).

Simulation of drug delivery via the IP route requires a model that is similar to the description of the drug transport equations of IV delivery, but requires unique source terms in the transport equations and different boundary conditions in the model formulation. Previous models in the area of peritoneal transport include pharmacokinetic compartmental models describing the transport processes occurring during peritoneal dialysis (Flessner et al., 1984; Stachowska-Pietka et al., 2006) and peritoneal chemotherapy (Flessner, 2005; Shah et al., 2009). Additionally, a theoretical model describing the IP transport of cisplatin was created (El-Kareh & Secomb, 2004) but no convective transport was taken into account. Similarly, a model comparing the IP and IV delivery of drugs was published (Winner et al., 2016), which also does not take into account the outward convective transport due to high IFP. A recent computational model described the transport of paclitaxel at three scales (i.e. tumor, IP cavity, whole organism) in a 2D pie-shaped tumor segment during IP chemotherapy (Au et al., 2014).

We present a fully 3D model to study drug transport in an isolated IP tumor nodule during IP chemotherapy. The model includes convective, diffusive and reactive drug transport in different tumor geometries and sizes and allows for testing the influence of changing therapy-related parameters (e.g. different types of drugs and tissue permeability) on the tissue penetration.

## Materials and methods

### Model geometry

The tumor nodules in peritoneal carcinomatosis have a large variety in shape and size. Therefore, three different geometries will be considered in this study.

**Figure 1.**
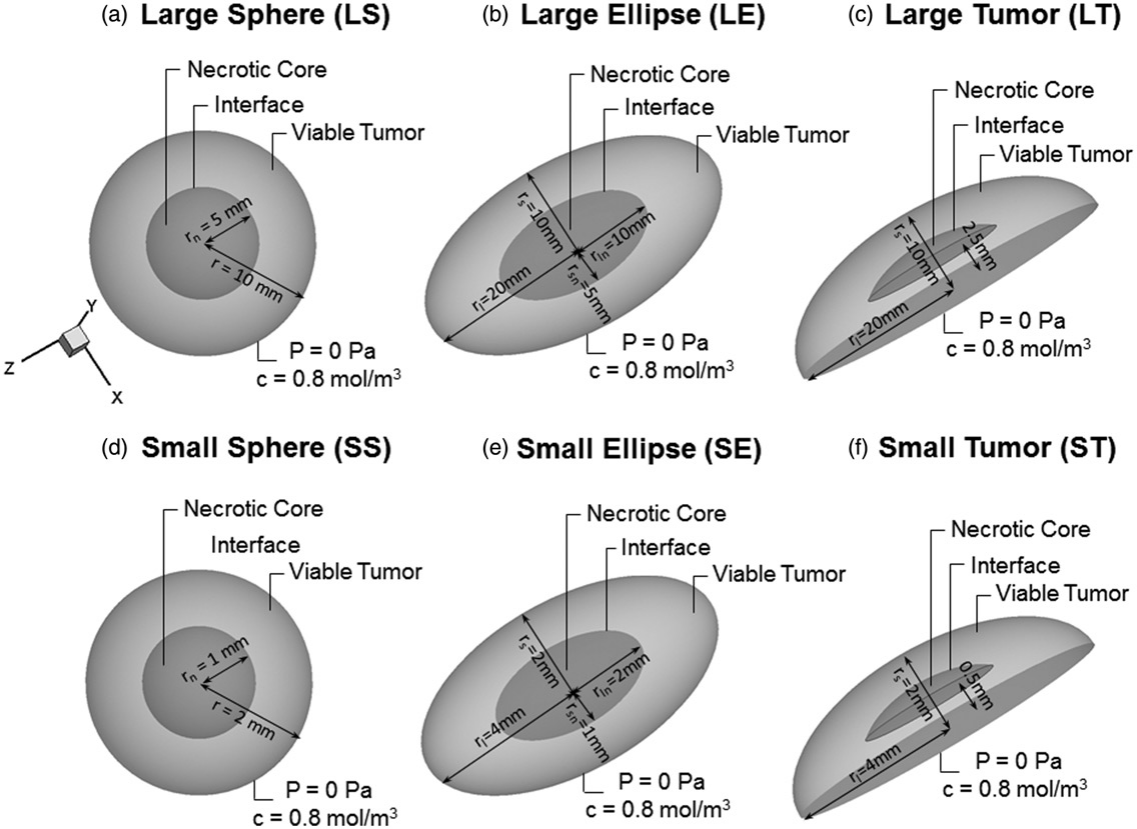
Visualization of the six used geometries in our model. (a and d) Geometries of spherical tumor shape comprising two different zones: a necrotic center of radius *r_n_* (darker gray area) and the viable tumor zone. A concentration and pressure boundary condition are applied at the outer edge of the tumor. (b and e) Geometries of an ellipsoid tumor shape. (c and f) Geometries of the peritoneal tumor shape.

The first geometry consists of a spherical tumor nodule with a radius r = 10 mm, labeled as LS (Figure 1a). The tumor is composed of two zones: a necrotic tumor zone (0 < *r* ≤ 5 mm), where neither living cells nor functional vascular or lymphatic system is present, and a viable tumor zone (5 < *r *≤ 10 mm), where living cells and a functional vasculature are present, but a functional lymphatic system is also lacking.

The second geometry, labeled as LE, has an ellipsoidal volume with the half length of the long axis (LA) (*r*_l_) and short axis (SA) (*r*_s_) equaling 20 and 10 mm, respectively. The necrotic zone is defined by 50% of the axis length of the viable tumor (i.e. 0 < *r*_ln_ ≤ 10 mm and 0 < *r*_sn_ ≤5 mm for the longer and smaller axis, respectively) (Figure 1b).

As a third geometry, the elliptic shape is cropped along the shorter axis, resulting in a more realistic tumor nodule geometry (LT) for a peritoneal metastasis. The length of the LA (*r*_l_) is kept at 20 mm, but the length in the perpendicular direction is reduced to 10 mm. The necrotic core in this case has a similar shape with the length of the longer axis (*r_l_*) being 8.7 mm and the length in the perpendicular direction being 4.53 mm (Figure 1c).

As clinical evidence suggests that patients with carcinomatosis of ovarian origin do not benefit from IP therapy if the nodules exceed a 10 mm diameter (Barakat et al., 2002), the same three geometries were also scaled down by a factor 5 (obtaining the geometries SS, SE and ST. respectively) to correlate the results with clinical data. All geometric properties are summarized in Figure 1(d)–(f).

### Governing equations

In this section, the equations describing the transport of the drug in the tumor tissue are presented based on seminal work by Baxter & Jain (1989).

#### IFP distribution

In a rigid porous medium like the interstitium, the momentum equation can be reduced to Darcy’s law (Bird et al., 2007):

(1) $$u=-K\nabla P_{i},$$

where u represents the interstitial fluid velocity (in m/s); K the conductivity of the tissue for interstitial fluid (m^2^/Pa s) and P_i_ the IFP (Pa). *K* is often defined in function of the dynamic viscosity of the fluid *μ* (Pa s) and the intrinsic permeability of the tissue *k* (m^2^):

(2) $$K=\frac{k}{\mu }.$$

The steady-state continuity equation for the incompressible interstitial fluid flow in normal tissue is given by (Bird et al., 2007):

(3) $$\nabla u=F_{v}-F_{l},$$

where ∇ represents the divergence operator; F_l_ a lymphatic drainage term for interstitial fluid (s^−1^) and F_v_ the fluid gain from the blood (s^−1^). Since there is a known lack of functional lymphatics in solid tumors, F_l_ = 0. The constitutive relation for F_v_ is based on Starling’s hypothesis (Baxter & Jain, 1989):

(4) $$F_{\text{v}}=\{\begin{array}{c} 0\;\;\text{for}\;r\leq r_{n},\\ \frac{L_{\text{p}}S}{V}\left(P_{\text{v}}-P_{\text{i}}-c\left(\pi_{\text{v}}-\pi_{\text{i}}\right)\right)\;\;\text{for}\;\;r>r_{n}, \end{array}$$

with *L*_p_ the hydraulic conductivity of the vasculature (m/Pa s), *S/V* the surface to volume ratio of the vasculature (m^−1^), *P*_v_ the vascular pressure (Pa), *P*_i_ the IFP (Pa), *c* the non-dimensional osmotic reflection coefficient, π_v_ the vascular osmotic pressure (Pa) and π_i_ the interstitial osmotic pressure (Pa). In this relation, a difference is made between the necrotic core (r ≤ r_n_ with *r_n_* the radius of the necrotic core), where no functional vasculature is present, and the viable tumor zone (r > r_n_) (Figure 1).

#### Species transport

Mass conservation of the drug is given by (Bird et al., 2007):

(5) $$\frac{\partial C_{\mathrm{drug}}}{\partial t}=D\nabla^{2}C_{\mathrm{drug}}-\nabla \left({\mathrm{uC}}_{\mathrm{drug}}\right)-S$$

with C_drug_ the time-dependent concentration of the drug present in the interstitium (mol/m^3^), D the diffusion coefficient (m^2^/s), ∇ ^2^ the Laplacian operator, ∇ the divergence operator and S the sink in drug concentration (mol/m^3^). The sink term S is in this work composed of two different terms:

(6) $$S=S_{\mathrm{bl}}+S_{\mathrm{cell}},$$

where S_bl_ represents the sink in the drug concentration related to the vascular uptake (mol/m^3^) and S_cell_ the sink in drug concentration due to cellular uptake (mol/m^3^). The closure term for the loss due to the cellular uptake of the drug is described by a first-order elimination, an approach commonly used in literature with β being a first-order elimination constant (s^−1^):

(7) $$S_{\mathrm{cell}}=C_{\mathrm{drug}}.$$

The closure term of the resorption by the vascular system finally is given by the following equation (Baxter & Jain, 1989):

(8) $$S_{\mathrm{bl}}=F_{v}\left(1-\sigma \right)C_{v}+\frac{P_{c}S}{V}(C_{v}-C_{i})\frac{{\mathrm{Pe}}_{v}}{e^{{\mathrm{Pe}}_{v}}-1}$$

with σ the reflection coefficient of vessels for the drug, C_v_ the concentration of drug in the vascular system (mol/m^3^), P_c_ the permeability of the vessel wall for the drug (m/s) and Pe_v_ the Péclet number that expresses the ratio of the mass transport contributions by convection to that by diffusion across the microvascular walls given by:

(9) $${\mathrm{Pe}}_{v}=\frac{F_{v}(1-\sigma )}{P_{c}S/V}.$$

Given that the tumor volume is low compared to the total body volume and the therapeutic time window is relatively small (typically 30 min to 1 h), the assumption is made that the vascular drug concentration remains negligible throughout the entire procedure. Given this assumption, Equation (4b) reduces to:

(10) $$S_{\mathrm{bl}}=-\frac{P_{c}S}{V}\;C_{i}\;\left(\frac{{\mathrm{Pe}}_{v}}{e^{{\mathrm{Pe}}_{v}}-1}\right).$$

### Baseline model

Because the time needed for tissue remodeling to occur is substantially larger than the therapeutic time scale, the outer boundary conditions for both the necrotic core and the tumor nodule are assumed to be constant throughout the simulation. At the interface between the two tumor zones (necrotic and viable zone), an interface boundary condition is imposed, implying continuity of all properties. On the edge of the tumor nodule, where the cytotoxic solution is in direct contact with the tumor tissue, a fixed drug concentration is maintained (i.e. 0.8 mol/m^3^) and the outlet pressure is set to 0 Pa. This zero outlet pressure boundary condition is a simplifications as, in reality, this pressure is likely to range anywhere between 0 and 20 Pa based on an abdominal surface area ranging between 1 and 2 m^2^, and an instillation fluid volume of 2 l (Nolph et al., 1990). This approximation was justified in this work by our observation that due to the high values of IFP in the nodules, small changes in this outlet pressure did not have a large influence on the results. All transport parameters are also being considered constant throughout the simulation and are summarized in Table 1.

**Table 1.** Parameters used for baseline simulations.

Parameter	Unit	Value	Reference
*r*	m	0.01	–
*r_*n*_*	m	0.005	–
*ρ*	kg/m^3^	1000	Teo et al. (2005)
*L*_p_	m/Pa s	2.10 × 10^−11^	Baxter & Jain (1989)
*K*	m^2^/Pa s	3.10 × 10^−14^	Baxter & Jain (1989)
*μ*	Pa s	1.00 × 10^−3^	Teo et al. (2005)
*K*	m^2^	3.10 × 10^−17^	Baxter & Jain (1989)
*S*/*V*	m^−1^	2.00 × 10^4^	Baxter & Jain (1989)
*P*_v_	Pa	2.08 × 10^3^	Baxter & Jain (1989)
*π*_b_	Pa	2.67 × 10^3^	Baxter & Jain (1989)
*π*_i_	Pa	2.00 × 10^3^	Baxter & Jain (1989)
*c*		0.82	Baxter & Jain (1989)
MW	g/mol	300	Shah et al. (2009)
*D*	m^2^/s	2.5 × 10^−10^	Shah et al. (2009)
*Β*	s^−1^	7.32 × 10^−4^	Shah et al. (2009)
σ		8.17 × 10^−5^	–
*P*_c_	cm/s	1.43 × 10^−4^	Shah et al. (2009)

### Parameter study

The baseline model presented in the previous section was then used to study drug diffusivity, the influence of vascular normalization therapy, the presence of a necrotic core and tissue permeability on the drug penetration.

Currently, a number of different drugs are used for IP chemotherapy. The influence of drug diffusivity on the penetration depth was studied to study whether a higher diffusivity resulted in an increase in penetration depth. For all baseline cases, drug-related parameters were taken from cisplatin (Table 1). We then compared results of these cisplatin baseline cases to cases where the drug diffusivity of paclitaxel was used. A summary of the values used for the different drugs can be found in Table 2; all values that are not reported in this table remain equal to those used in the baseline cases.

**Table 2.** Parameter values used to study the influence of several transport-related parameters.

	*S*/*V* (m^−1^)	*L*_p_ (m/Pa s)	*C* (–)	Reference
Vascular normalization simulations				
Baseline values	2.00 × 10^4^	2.10 × 10^−11^	0.82	Baxter & Jain (1989)
50% Vascular normalization	1.35 × 10^4^	1.19 × 10^−11^	0.865	–
100%Vascular normalization	7.00 × 10^3^	2.70 × 10^−12^	0.91	Baxter & Jain (1989)
	Diffusion coefficient (m^2^/s)	Reference	IC_50_ [mol/m^3^]	
Drug diffusion simulations				
Cisplatin	2.5 × 10^−10^	Shah et al. (2009)	6.2 × 10^−3^	De Vlieghere et al. (2016)
Paclitaxel	0.77 × 10^−10^	Winner et al. (2016)	1.4 × 10^−6^	Smith et al. (2005)
		Intrinsic permeability (m^2^)		
Permeability simulations				
Normal tissue		6.4 × 10^−18^		Baxter & Jain (1989)
Commonly used value		3.1 × 10^−17^		Baxter & Jain (1989)
Lower limit of the range		6.4 × 10^−17^		–

All parameters that are not listed in this table are kept at their baseline value (Table 1) for each simulation.

Certain vascular normalization therapies have been shown to normalize the architecture and permeability of the microvasculature, resulting in a lower IFP and better drug distribution (Fukumura & Jain, 2007). Recently, works by Shah et al. (2009) and Gremonprez et al. (2015) showed similar improvement in drug penetration after vascular normalization therapy for IP chemotherapy. We attempted to mimic vascular normalization to test whether our model would be able to reproduce the results from these works. In the first step, all vascular-related properties (*L*_p_; S/V; *c*) were interpolated halfway between typical tumor tissue and normal tissue values. In the second step, full normalization of all vascular-related parameters was simulated (Table 2) (Baxter & Jain, 1989). Results of these two cases will then be compared to their respective baseline values.

The presence of necrotic regions in solid tumors is well documented but the exact size and location of the necrotic core is not always known. In order to estimate the impact of this uncertainty on our model, we omitted the necrotic core that was implemented in the baseline case by setting the conditions in this region equal to those of viable tumor tissue, and compared the resulting pressure profiles and penetration depths.

Due to the differences in ECM, cell density and the presence of CAFs, the tumor tissue permeability is likely to be significantly different from the healthy surrounding tissue permeability. As tissue permeability is notoriously difficult to quantify, no reliable values are currently present in literature, and most models use an arbitrary tenfold of the healthy tissue value (Baxter & Jain, 1989). The influence of this assumption on the penetration depth of the drug was investigated by varying the permeability and comparing the penetration depth to the baseline cases. A summary of the permeability range used can be found in Table 2.

### Numerical methods

All geometries were created and meshed in COMSOL multiphysics (COMSOL, Inc., Burlington, VT). All equations mentioned in the methods section were also implemented in COMSOL. A segregated approach was used for solving the continuity, momentum transport and mass transport equations. All cases were run both in steady state and as a transient model. Due to the length of the IP therapy procedure (typically ranging from 30 min to 1 h), a time resolution of 30 s was chosen when transient simulations were performed. As a convergence criterion, a drop of 4 orders of magnitude in the residuals was chosen.

### Analyzed variables

**Figure 2.**
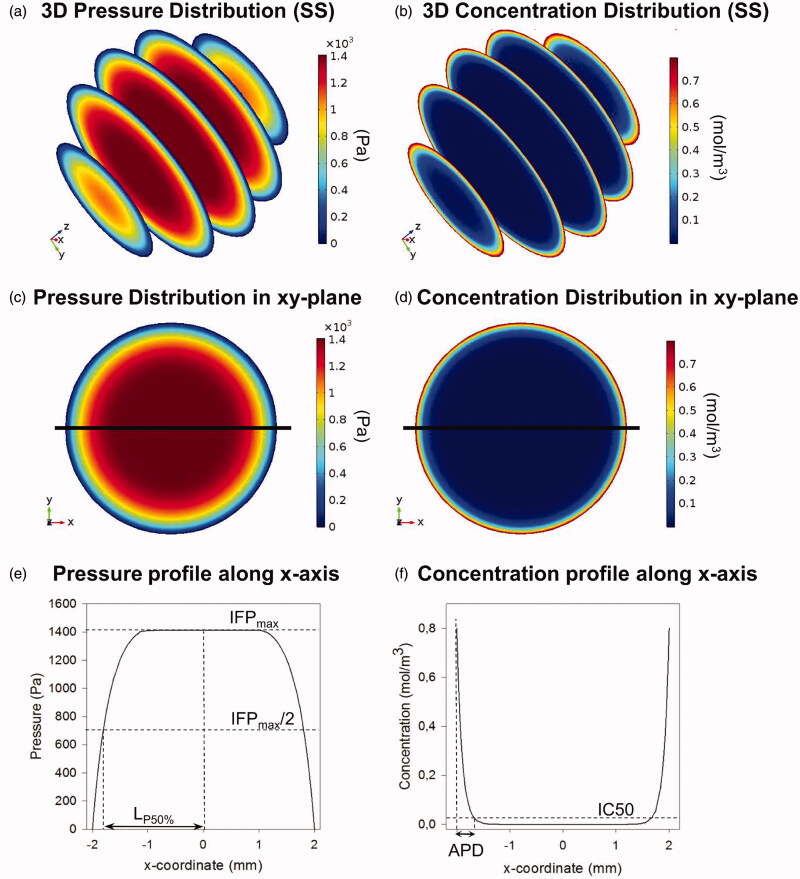
Summary of model output and analyzed variables. (a and b) Three-dimensional pressure and concentration distributions in the small spherical geometry (SS). (c and d) Two-dimensional pressure and concentration distributions in the *xy*-plane of the SS geometry. The *x*-axis is plotted on the figures in black. (e and f) One-dimensional pressure and concentration profiles along the *x*-axis in the SS geometry. All analyzed variables as discussed in the “Analyzed variables” section are presented in the figure.

For all simulations, pressure and concentration profiles were analyzed along either the *x*-axis (spherical geometries) or along both *x*- and *z*-axis (ellipsoid and tumor geometries) (Figure 2c and d). We will characterize the pressure profile by the maximal IFP (IFP_max_) and the steepness of the profile (Figure 2e). Steepness is characterized by the LP50 value, which we define as the distance starting from the tumor center over which the pressure drops to 50% of its maximal value. The steeper the pressure profile, the higher the LP50 value will be.

From the concentration profiles along the axis, the penetration depth is determined. Penetration depth is represented in this work by two different metrics. On the one hand, absolute penetration depths (APDs) are reported, defined as the maximal depth along the axis of interest where the drug concentration exceeds the corresponding half maximal inhibitory concentration IC_50_ value of the drug (Figure 2f). The second metric is the relative penetration depth percentage (PD%), representing the percentage of the radius where concentration values exceed the corresponding IC_50_ value of the drug used.

During IP chemotherapy, the diffusive and convective drug transport happens in different directions: the diffusive transport is directed inwards into the tumor, whereas the convective transport is directed outwards out of the tumor. The penetration depth of the drug will be determined by the relative influence of both contributions. In the study of transport phenomena, the Péclet number (Pe) is a dimensionless number that expresses the ratio of the mass transport contributions by convection to that by diffusion of the drug into the tumor tissue. In the context of mass transport, it is defined as:

(11) $$\mathrm{Pe}=\frac{L\cdot u}{D}$$

with *L* the characteristic length of the system (m), *u* the maximal velocity magnitude (m/s) and *D* the diffusion coefficient of the drug (m^2^/s).

## Results

### Baseline cases

As illustrated in Figure 2, the simulations allow us to determine 3D pressure and concentration distributions in all geometries.

**Figure 3.**
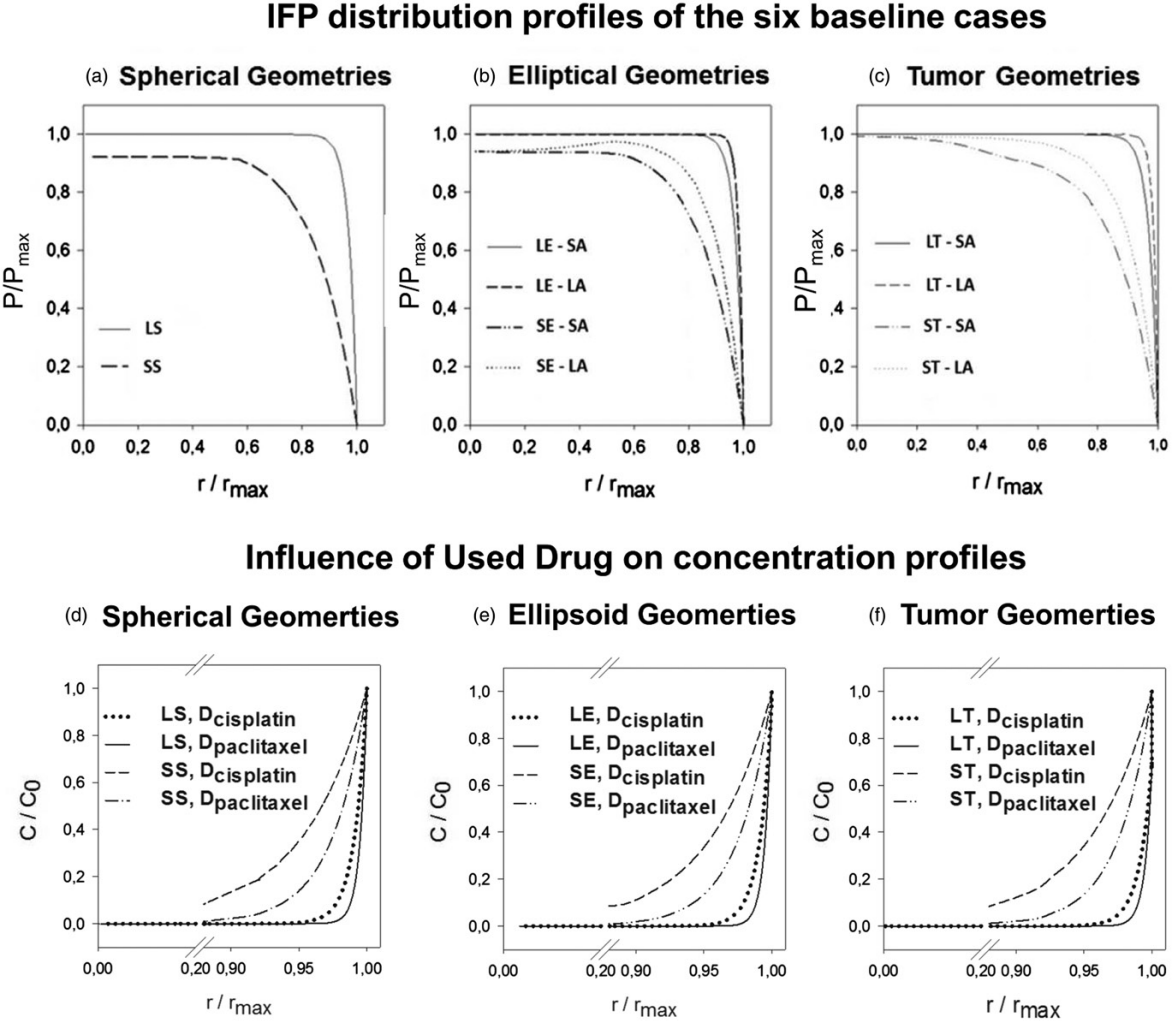
(a–c) Interstitial fluid pressure (IFP) distribution profiles of the six baseline cases. Both length along the axis and IFP are normalized; the former with respect to the maximal length along the axis, the latter with respect to the overall maximal pressure (IFP_max_ = 1533.88 Pa). The figures (d–f) show a comparison between the resulting concentration profiles after IP chemotherapy in which cisplatin or paclitaxel is used. Concentrations are normalized with respect to the boundary concentration (C_0 _=_ _0.8 mol/m^3^).

The maximal IFP reached in the model was 1533.88 Pa (11.5 mmHg), and this value was reached in all three large geometries. The shape of the pressure profiles, however, differed between these three cases with the least steep profile being the one for the SA of the ST geometry (LP50 = 0.89) (Figure 3c) and most steep for the LA of the LE geometry (LP50 = 0.99) (Figure 3b). Overall, the steeper the pressure profile, the higher the maximal interstitial fluid velocity, and therefore the higher the radial outward convective flow will be.

APD ranged from 0.36 mm (SA-LT) to 0.49 mm (LA-ST) with the corresponding PD% ranging from 1.81% to 21.29% (Supplementary Appendix 1). Furthermore, the IFP was found to be consistently lower and penetration depths higher in all smaller sized geometries (Figure 3). In the elliptical and tumor geometries, a shape effect could be noted, resulting in different APDs and PD%s along the SA and LA.

### Drug type

When comparing the pressure profiles characteristics, it is clear that the IFP_max_ and LP50 do not change in any of the cases (Supplementary Appendix 1). Concentrations of cisplatin are consistently higher in all geometries, at all points (Figure 3d–f). However, due to the large difference in IC_50_ values between different drugs (Table 2), the APD and PD% are higher for paclitaxel when compared to cisplatin (Supplementary Appendix 1). APD ranged from 0.54 mm (SA-LE) to 0.75 mm (SA-ST) for paclitaxel versus 0.36 mm (LA-LE) to 0.49 mm (SA-ST) for cisplatin. In general, smaller geometries showed a larger improvement in penetration depth (Supplementary Appendix 1). As changes in the diffusion coefficient only influence the diffusive transport of the drug, all pressure profiles were equal to the ones found in the corresponding baseline cases.

### Vascular normalization

The effect of vascular normalization therapy during IP chemotherapy on the IFP_max_, LP50, APD en PD% was simulated in two steps (50% vascular normalization and 100% vascular normalization) (Table 2). We found that vascular normalization lowered the IFP in all cases and decreased the steepness of all pressure profiles. The lowest IFP_max_ was reached in the case of the small spherical geometry (IFP_max_ = 259.4 Pa) and the lowest LP50 was obtained along the SA of the small tumor geometry (LP50 = 0.70) (Supplementary Appendix 1).

**Figure 4.**
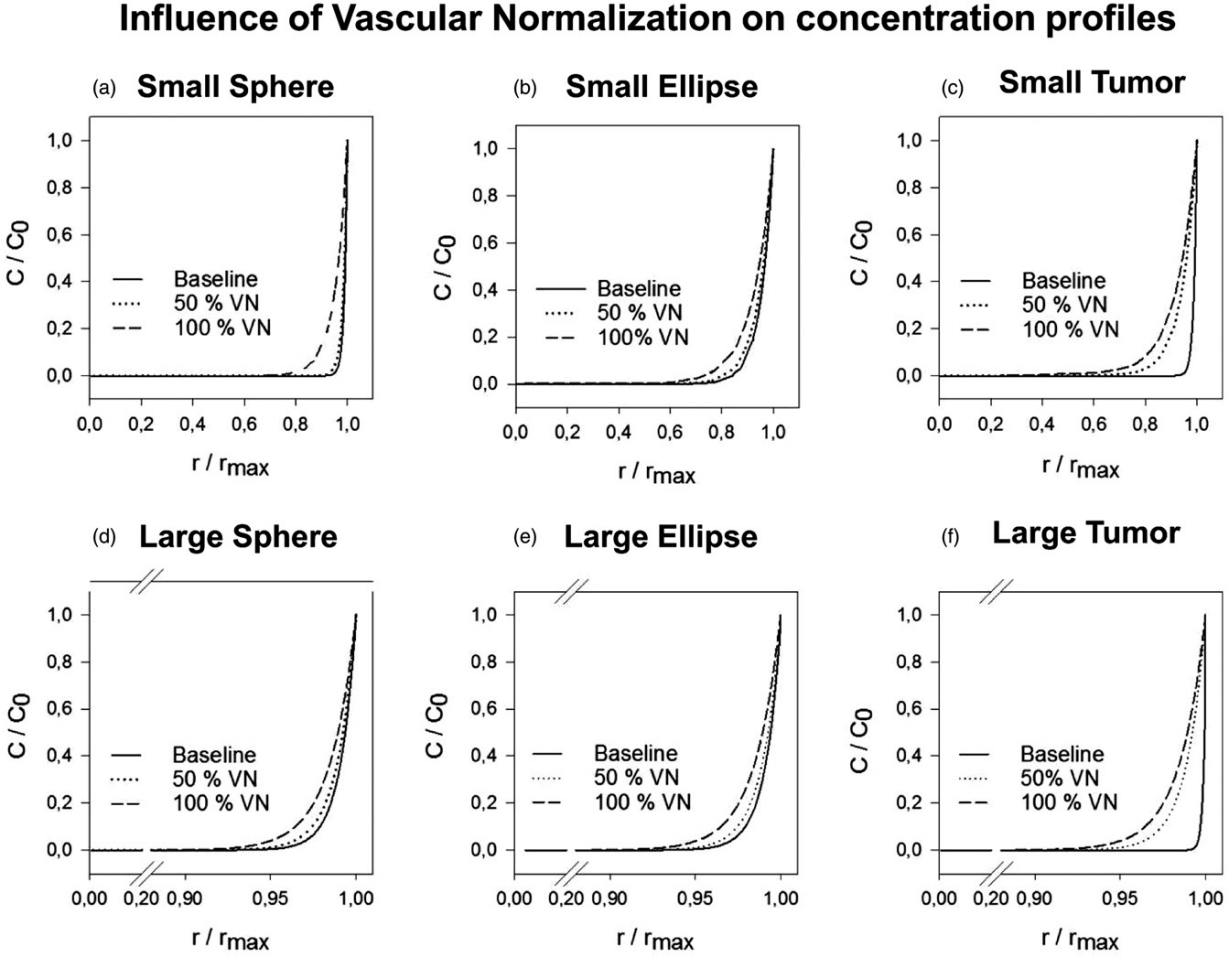
Normalized concentration profiles in which both length along the axis and concentration are normalized; the former with respect to the maximal length along the axis, the latter with respect to the boundary concentration (*C*_0 _=_ _0.8 mol/m^3^). The figures (a–f) show the resulting concentration profiles after vascular normalization therapy for all geometries.

Results of the 100% vascular normalization simulation in the small tumor showed a penetration depth exceeding half of the tumor radius (PD% = 51.64, absolute penetration = 1.0 mm) along the SA (Figure 4c). In general, vascular normalization showed a nonlinear, positive effect on the penetration depth in all cases (Supplementary Appendix 1). APDs ranged from 0.45 to 0.58 mm for 50% vascular normalization and between 0.65 and 1.03 mm for 100% vascular normalization. Relative improvements with respect to the respective baseline PD% ranged from 0.39% (LA-LE) to 7.34% (SA-SE) for the 50% normalization and between 0.69% (LA-LT) to 29.35% (SA-ST) for 100% vascular normalization.

### Necrotic core

Omitting the necrotic core from the computation and thus describing the tumor as a single homogeneous zone increased the IFP_max_ in all three smaller geometry cases. In the larger geometries, a further increase was not possible and therefore IFP_max_ remained the same. The maximal interstitial fluid velocities (IFV_max_) were, however, lower in most cases without necrotic core when compared to the baseline cases, with the exception of the small ellipse and tumor geometry. Overall, the effect on the penetration depth was limited with differences in PD% never exceeding 3.56% (SA-SE) (Supplementary Appendix 1).

### Permeability

Tissue permeability marginally influences penetration depth. Overall lower permeabilities lead to lower IFV_max_ and higher IFP_max_, however, no measurable differences larger than 0.19% could be noted in relative penetration depth (Supplementary Appendix 1).

## Discussion and conclusion

In this study, a 3D CFD model of a peritoneal tumor nodule was developed to study the mass transport of drugs during IP chemotherapy. The model is, to our knowledge, the first fully 3D parametrical model that studies the influence of different parameters on the penetration depth of drugs during IP chemotherapy. Baseline cases of the model are presented for two different sizes of three different geometries. The model was used to study the influence of vascular normalization therapy, drug diffusivity, the presence of a necrotic core and tissue permeability on the drug penetration.

When comparing our results of the baseline cases with those obtained in previous studies, it was found that APD in all baseline cases ranged from 0.36 to 0.49 mm, which is in good agreement with the experimentally defined range of 0.41–0.56 mm where carboplatin (another platinum-based drug of roughly the same size) was used (Ansaloni et al., 2015). One of the main findings of the baseline cases was the profound effect of the tumor size on the drug penetration depth. When averaged over all baseline cases, the smaller tumors were shown to have higher PD% (PD%_average_ = 17.83% for tumors with a diameter/characteristic length of 2–4 mm) than the larger ones (PD%_average_ = 3.24% for tumors with a diameter/characteristic length of 10–20 mm). These findings are consistent with the results obtained by Barakat et al. (2002) and Ansaloni et al. (2015), stating that tumor nodules with a radius larger than 10 and 2.5 mm, respectively, do not benefit from IP chemotherapy. Hence, there seems to be a critical size of tumor nodules that are responsive to IP treatment, showing the importance of removing nodules of larger sizes before the onset of IP chemotherapy. In this work, we focused on vascular tumors. If no vasculature is present, the IFP will not build-up in the tumor tissue, thereby eliminating the large outward convective flow that limits the inward diffusion of the drug. Additionally, no drug will be resorbed through the tumor vasculature and subsequently lost for further transport. Given these two differences, the outward flow and blood sink terms are canceled in Equation (5) and the model would predict full penetration of the drug regardless of the tumor size, given sufficient time.

During IP chemotherapy, the diffusive and convective drug transport occurs in different directions: the diffusive transport is directed inwards into the tumor, whereas the convective transport is directed outwards out of the tumor. The Péclet number gives an idea of the relative influence of these two opposing modes of transport. In all baseline cases, Péclet numbers are larger than one (1.09–22) and therefore convective transport will be dominant over diffusive transport. The convective transport is governed by the pressure differences inside the tumor and this work showed that smaller tumors had consistently lower IFP_max_ (IFP_max, av _= 1477.1 Pa) than larger ones (IFP_max, av _= 1533.9 Pa) (Figure 3). In agreement, Ferretti et al. (2009) measured higher IFP’s for tumor nodules of larger sizes (typically a diameter in the range of 10–20 mm) grown from the same cell lines. We can therefore account the lower penetration depths in the larger geometries (Pe = 1.09–10.52) to the increase in the outward convective flow when compared to the smaller geometries (Pe = 6.7–22).

The demonstrated shape effect on the penetration depth (Figure 3a–c) illustrates the added value of using fully 3D models. Spherical tumors had uniform radial penetration, whereas elliptical tumors had different penetration depths along different axes (i.e. deeper percentual penetration along the SA; for example Large Ellipsis: PD%=3.77 (SA) − PD%=1.96 (LA)). The tumor with its flat side, mimicking the contact area with the peritoneum, had an even more pronounced shape effect [e.g. Large Tumor: PD%=3.82 (SA) − PD%=1.81(LA)] (Supplementary Appendix 1).

We also compared CFD results for the penetration depth of two commonly used drugs in IP treatment, that is cisplatin and paclitaxel. In the model, the difference between the two drugs is primarily reflected in a different diffusion coefficient, which is much higher for cisplatin. As such, when using the same drug dose/boundary conditions, concentrations were consistently higher for cisplatin in all geometries at all times (Figure 3d–f). The drug’s IC_50_ values, however, are substantially different (i.e. the IC_50_ value of cisplatin is a factor 4500 larger than the IC_50_ of paclitaxel; see also the “Parameter study” section). As the penetration depth as defined in this work uses the IC_50_ value as the cutoff between zones with and without significant drug penetration, this large difference in IC_50_ values translates into a higher APD and PD% for paclitaxel when compared to cisplatin (Supplementary Appendix 1). APD for paclitaxel ranged from 0.54 mm (LA-SA) to 0.75 mm (ST-SA) and APD for cisplatin ranged from 0.36 mm (SA-LT) to 0.49 mm (LA-ST). Nonetheless, it should be noted that in these simulations, the same boundary concentration of 0.8 mol/m^3^ was used for both drugs, which is a value that is equivalent to a used dose of 120 mg/m^2^ cisplatin, but does not correspond with the clinical practice for paclitaxel. Therefore, an additional case was set-up in a spherical geometry with a 10 mm radius and a paclitaxel boundary concentration of 0.14 mol/m^3^, which corresponds with a clinical dose of 60 mg/m^2^ (Kampan et al., 2015).The APD of paclitaxel was 0.64 mm in this case which is, when compared to the penetration depth of cisplatin used at the clinical dose (APD = 0.40 mm), still an improvement.

Note that the IC_50_ values are taken from *in vitro* analysis and protein-binding in an *in vivo* setting might influence these values (Zeitlinger et al., 2011). Therefore, we found that when considering different drugs to use for IP chemotherapy, not only transport parameters, but also biological parameters like IC_50_ values and protein binding should be taken into account.

When applying different degrees of vascular normalization to our model, we found significant improvements in drug penetration with APD ranging from 0.45 to 0.70 mm in the larger tumors and from 0.52 to 1.03 mm in the smaller tumors when compared to the baseline range of 0.36 to 0.49 mm. The relationship between high IFP in solid tumors and the abnormal microvasculature has been well documented in literature (Heldin et al., 2004). The tumor blood vessel’s leakiness and irregular shape lead to the excessive ultrafiltration of interstitial fluid, which is one of the contributing factors of high IFP. We then compared our *in silico* data with the recent experimental data, in which IP tumors were pretreated with several different VEGF(R) inhibitors to normalize the microvasculature before subjecting them to IP chemotherapy (Gremonprez et al., 2015). Tumor size was 124.85 mm^3^ on average, which would be equivalent to a spherical tumor with a 5 mm radius and pre-treating the tumors with certain VEGF(R) inhibitors decreased the IFP. An increased platinum concentration, measured by laser ablation inductively coupled mass spectrometry (LA-ICP-MS), was detected in the peritoneal border area (up to 1.68 mm) of the pretreated tumors with lower IFP. When a detection limit similar to the one of LA-ICP-MS is applied to the concentration profile obtained after 50% and 100% vascular normalization in a 10 mm radius sphere, we found APDs of 1.6 mm and 2.1 mm, respectively, which is in good agreement with experimental data (Gremonprez et al., 2015).

Interestingly, explicit modeling of a central necrotic core in the tumor had little influence on the penetration depth [i.e. maximal APD difference was 0.05 mm (SE-LA)]. These results are important, as the existence of a necrotic core is a well described property of solid tumors, but little is known about the shape, size and even the number of necrotic regions. Our work shows that the inaccuracy in the model due to the uncertainty about the necrotic core will be relatively small (max. 3.56 PD%). It is likely that – due to the different drug sources in IV and IP therapy (the vascular network and the outer edge of the tumor, respectively) – these results are unique for the case of IP drug delivery, and cannot be extrapolated toward IV chemotherapy.

The variation of the intrinsic tissue permeability over an order of magnitude did not affect penetration depth significantly (rel PD% < 0.19%). We calculated the *Pe* number for each simulated case in order to determine a possible cause for this effect. We found an increase in IFP_max_ and a decrease in IFV_max_ when permeabilities were lower. Overall, Péclet numbers ranged from 0.6 to 348.7. *Pe* values below 1 were noted in some cases, indicating that the diffusive transport is likely to become the dominant transport phenomenon in very low permeability cases. All these findings, however, did not translate to quantifiable differences in APD, suggesting that the model might be fairly robust toward changes in permeability.

Although it might not replace *in vivo* experiments, this parametrical CFD model for IP chemotherapy is a powerful tool that allows us to gain insight in how much influence certain parameters have on the penetration depth of the drug. It is to our knowledge the first parametrical CFD model in the context of IP chemotherapy that allows for the prediction of drug penetration depths. Furthermore, the model could be used to reproduce a number of literature validated trends (e.g. effect of tumor size on penetration depth and effect of vascular normalization therapy) and the calculated penetration depths proved a good match to the ones found in literature.

A number of assumptions were made when developing the numerical models. For example, in this work drug concentration at the outer edge of the tumor is fixed during the entire simulation due to unavailability of suitable experimental data. We aim to sample IP fluid during the IP chemotherapy in follow-up validation experiments in order to implement a more accurate inlet concentration boundary condition. Moreover, the entire model treats the vasculature as a distributed source without taking into account the, sometimes large, spatial heterogeneities within tumors. Implementing more realistic boundary conditions for the concentration at the outer edge of the tumor and allowing for the spatial variation of vascular properties, would further improve the model. Another limitation of the model is the lack of sink terms implemented that represent the effect of other physiological phenomena such as ECM binding and plasma protein binding.

Future work will include the validation of the model in an animal model of peritoneal carcinomatosis. Given the importance of tumor size and the noted shape effect, MRI images of the tumor nodules will be used to segment the actual tumor geometries.

In conclusion, a parametrical 3D CFD model was developed for the drug mass transport in a single tumor nodule during IP chemotherapy. Tumors of smaller sizes respond better to treatment when compared to larger ones. Vascular normalization therapy lowered the IFP and steepness of pressure profiles, thereby increasing drug penetration depths. When selecting a drug for IP therapy, not only transport properties should be taken into account, but also biological properties (e.g. IC_50_-value), as these may have an influence on the therapeutic outcome. Furthermore, both modeling of the necrotic core and the intrinsic tissue permeability had a limited effect on the predicted penetration depth.

## Supplementary Material

Appendix1_DD.docx

## Notes

**Supplementary material available online**
