# Supplementary material for: Mathematical modeling of intraperitoneal drug delivery: simulation of drug distribution in a single tumor nodule
Source: Drug Deliv. 2017 Feb 9;24(1):491–501. doi: 10.1080/10717544.2016.1269848 (PMC8240979; doi:10.1080/10717544.2016.1269848)
Supplement: Appendix1_DD.docx [file IDRD_A_1269848_SM5986.docx]

| **Appendix 1: Characteristics of all simulations** | | | | | | |
| --- | --- | --- | --- | --- | --- | --- |
| **Geometry** | **Characteristic** | **IFPmax** | **LP50** | **APD** | **PD%** | **Difference in** |
|  |  | **[Pa]** | **[mm]** | **[mm]** | **[-]** | **PD%** |
| **Baseline cases** | | | | | | |
| LS | Baseline Case | 1533.9 | 0.98 | 0.40 | 4.04 | - |
| SS | Baseline Case | 1413.3 | 0.89 | 0.42 | 20.82 | - |
| LE | Baseline Case | 1533.9 | 0.98(SA)/ 0.99(LA) | 0.38(SA)/0.39(LA) | 3.77(SA)/1.96(LA) | - |
| SE | Baseline Case | 1495.4 | 0.90(SA)/ 0.93(LA) | 0.38(SA)/0.47(LA) | 21.39(SA)/11.64(LA) | - |
| LT | Baseline Case | 1533.9 | 0.98(SA)/0.99(LA) | 0.38(SA)/0.36(LA) | 3.82(SA)/ 1.81(LA) | - |
| ST | Baseline Case | 1522.5 | 0.89(SA)/0.95(LA) | 0.45(SA)/0.49(LA) | 21.29(SA)/12.31(LA) | - |
| **Effect of vascular normalization** | | | | | | |
| LS | 50%VN | 1503.8 | 0.97 | 0.49 | 4.85 | 0.81 |
| LS | 100%VN | 1379.3 | 0.91 | 0.70 | 6.99 | 2.95 |
| SS | 50%VN | 1078.4 | 0.87 | 0.53 | 26.71 | 5.89 |
| SS | 100%VN | 259.4 | 0.84 | 0.78 | 38.76 | 17.95 |
| LE | 50%VN | 1503.9 | 0.97/0.98 | 0.45/0.47 | 4.52(SA)/2.35(LA) | +0.75(SA)/+0.39(LA) |
| LE | 100%VN | 1446.7 | 0.91/0.94 | 0.65/0.68 | 6.53(SA)/3.39(LA) | +2.76(SA)/+1.43(LA) |
| SE | 50%VN | 1266.4 | 0.87/0.90 | 0.53/0.57 | 26.43 (SA)/14.20(LA) | +5.04 (SA)/+2.56 (LA) |
| SE | 100%VN | 370.6 | 0.84/0.84 | 0.72/0.89 | 35.82(SA)/22.33(LA) | +14.43(SA)/10.69(LA) |
| LT | 50%VN | 1503.9 | 0.97/0.98 | 0.46/0.50 | 4.58(SA)/2.50(LA) | +0.76(SA)/+0.69(LA) |
| LT | 100%VN | 1466.4 | 0.90/0.94 | 0.66/0.65 | 6.60(SA)/3.28(LA) | +2.78(SA)/+1.47(LA) |
| ST | 50%VN | 1389.8 | 0.83/0.88 | 0.52/0.58 | 25.82(SA)/14.42(LA) | +3.53(SA)/+2.10(LA) |
| ST | 100%VN | 531.1 | 0.70/0.77 | 1.03/0.74 | 51.64(SA)/18.50(LA) | +29.35(SA)/+6.4(LA) |
| **Effect of drug diffusivity** | | | | | | |
| LS | Paclitaxel | 1533.9 | 0.98 | 0.64 | 6.38 | 2.33 |
| SS | Paclitaxel | 1413.3 | 0.89 | 0.68 | 33.85 | 13.03 |
| LE | Paclitaxel | 1533.9 | 0.98(SA)/ 0.99(LA) | 0.54/0.56 | 5.44(SA)/2.82(LA) | +1.67 (SA)/+0.86 (LA) |
| SE | Paclitaxel | 1495.4 | 0.90(SA)/ 0.93(LA) | 0.67/0.70 | 33.25(SA)/17.48(LA) | +11.86(SA)/+5.85(LA) |
| LT | Paclitaxel | 1533.9 | 0.98(SA)/0.99(LA) | 0.66/0.60 | 6.60(SA)/3.00(LA) | +2.79(SA)/+1.19(LA) |
| ST | Paclitaxel | 1522.5 | 0.89(SA)/0.95(LA) | 0.75/0.74 | 37.50(SA)/18.49(LA) | +15.21(SA)/+6.17(LA) |
| **Effect of necrotic core** | | | | | | |
| LS | no NC | 1533,9 | 0.98 | 0,41 | 4,13 | 0.09 |
| SS | no NC | 1519,5 | 0.89 | 0,44 | 21,89 | 1.08 |
| LE | no NC | 1533,9 | 0.98(SA)/0.99(LA) | 0,42/0,41 | 4,15(SA)/2,05(LA) | +0,38(SA)/+0,09 (LA) |
| SE | no NC | 1526,3 | 0.91(SA)/0.93(LA) | 0,36/0,42 | 17,83(SA)/10,58(LA) | -3,56(SA)/-1,06(LA) |
| LT | no NC | 1533,9 | 0.98(SA)/0.99(LA) | 0,40/0,37 | 4,01(SA)/1,83(LA) | +0,20(SA)/ 0,02 (LA) |
| ST | no NC | 1526,5 | 0.90(SA)/0.94(LA) | 0,44/0,48 | 22,12(SA)/11,84(LA) | -0,17(SA)/-0,48 (LA) |
| **Effect of intrinsic permeability** | | | | | | |
| LS | k = 6.4 · 10-17 | 1533.9 | 0.98 | 0.40 | 4.04 | 0 |
| LS | k = 3.1 · 10-17 | 1533.9 | 0.98 | 0.40 | 4.04 | - |
| LS | k = 6.4 · 10-18 | 1533.9 |  | 0.37 | 3.69 | -0.35 |
| SS | k = 6.4 · 10-17 | 1206.4 | 0.89 | 0.42 | 20.82 | 0 |
| SS | k = 3.1 · 10-17 | 1413.3 | 0.89 | 0.42 | 20.82 | - |
| SS | k = 6.4 · 10-18 | 1532.2 | 0.89 | 0.42 | 20.82 | 0 |
| LE | k = 6.4 · 10-17 | 1533.9 | 0.97(SA)/0.98(LA) | 0.39(SA)/0.41(LA) | 3.91(SA)/2.07(LA) | +0.14(SA)/+0.11(LA) |
| LE | k = 3.1 · 10-17 | 1533.9 | 0.98(SA)/ 0.99(LA) | 0.38(SA)/0.39(LA) | 3.77(SA)/1.96(LA) | - |
| LE | k = 6.4 · 10-18 | 1533.9 | 0.98(SA)/0.99(LA) | 0.38(SA)/0.38(LA) | 3.77(SA)/1.89(LA) | 0(SA)/-0.07(LA) |
| SE | k = 6.4 · 10-17 | 1256.4 | 0.88(SA)/0.91(LA) | 0.43(SA)/0.47(LA) | 21.39(SA)/11.65(LA) | 0(SA)/+0.01(LA) |
| SE | k = 3.1 · 10-17 | 1495.4 | 0.90(SA)/ 0.93(LA) | 0.38(SA)/0.47(LA) | 21.39(SA)/11.64(LA) | - |
| SE | k = 6.4 · 10-18 | 1533.9 | 0.90(SA)/ 0.93(LA) | 0.38(SA)/0.47(LA) | 21.39(SA)/11.64(LA) | 0(SA)/0(LA) |
| LT | k = 6.4 · 10-17 | 1533.9 | 0.97(SA)/0.98(LA) | 0.38(SA)/0.38(LA) | 3.82(SA)/1.90(LA) | 0(SA)/+0.09(LA) |
| LT | k = 3.1 · 10-17 | 1533.9 | 0.98(SA)/0.99(LA) | 0.38(SA)/0.36(LA) | 3.82(SA)/ 1.81(LA) | - |
| LT | k = 6.4 · 10-18 | 1533.9 | 0.99(SA)/0.99(LA) | 0.37(SA)/0 | 3.70(SA)/1.81(LA) | -0.12(SA)/0(LA) |
| ST | k = 6.4 · 10-17 | 1346.0 | 0.89(SA)/0.92(LA) | 0.45(SA)/0.50(LA) | 22.29(SA)/12.50(LA) | 0(SA)/+0.19 |
| ST | k = 3.1 · 10-17 | 1522.5 | 0.89(SA)/0.95(LA) | 0.45(SA)/0.49(LA) | 22.29(SA)/12.31(LA) | - |
| ST | k = 6.4 · 10-18 | 1533.9 | 0.85(SA)/0.90(LA) | 0.45(SA)/0.49(LA) | 22.29(SA)/12.30(LA) | 0(SA)/-0.01(LA) |

Appendix 1: Characteristics of all simulations. Maximal interstitial fluid pressures (IFP_max_ ) and distances necessary for the pressure to drop to 50% of its maximal value (LP50) are stated to characterize the pressure profile, absolute penetration depth (APD) and penetration depth percentages (PD%) are given to characterize the concentration profile. The stated differences in PD% are always with respect to the corresponding baseline case. A summary of all used geometries and their corresponding abbreviation can be found in Figure 1. **a** Characteristics of the six baseline cases. **b** Characteristics of the vascular normalization (VN) simulations. **c** Characteristics of the drug diffusivity simulations. **d** Characteristics of the simulations with and without necrotic core (NC). **e** Characteristics of simulations with varying intrinsic permeability (k).
